# Te Vacancy Defect Engineering on Fe_3_GeTe_2_ (001) Basal Planes for Enhanced Oxygen Evolution Reaction: A First-Principles Study

**DOI:** 10.3390/nano15161272

**Published:** 2025-08-18

**Authors:** Yunjie Gao, Wei Su, Yuan Qiu, Dan Shan, Jing Pan

**Affiliations:** 1 School of Electronic Engineering, Yangzhou Polytechnic University, Yangzhou 225009, China; 101566@yzpc.edu.cn (Y.G.);; 2 College of Physics Science and Technology, Yangzhou University, Yangzhou 225002, China; jssuwei@126.com; 3 School of Electronic and Information Engineering/Carbon Based Low Dimensional Semiconductor Materials and Device Engineering Research Center of Jiangsu Province, Yangzhou Polytechnic Institute, Yangzhou 225127, China

**Keywords:** Te vacancies, first-principles density functional theory (DFT), oxygen evolution reaction (OER), overpotential

## Abstract

Photocatalytic water splitting for hydrogen production is an attractive renewable energy technology, but the oxygen evolution reaction (OER) at the anode is severely constrained by a high overpotential. The two-dimensional vdW ferromagnetic material Fe_3_GeTe_2_, with its good stability and excellent metallic conductivity, has potential as an electrocatalyst, but its sluggish surface catalytic reactivity limits its large-scale application. In this work, we adapted DFT calculations to introduce surface Te vacancies to boost OER performance of the Fe_3_GeTe_2_ (001) surface. Te vacancies induce the charge redistribution of active sites, optimizing the adsorption and desorption of oxygen-containing intermediates. Consequently, the overpotential of the rate-determining step in the OER process of Fe_3_GeTe_2_ is reduced to 0.34 V, bringing the performance close to that of the benchmark IrO_2_ catalyst (0.56 V). Notably, the vacancies’ concentration and configuration significantly modify the electronic structure and thus influence OER activity. This study provides important theoretical evidence for defect engineering in OER catalysis and offers new design strategies for developing efficient and stable electrocatalysts for sustainable energy conversion.

## 1. Introduction

Driven by environmental pollution and energy security challenges, the global transition toward clean energy is accelerating [1,2]. Among the alternatives, hydrogen energy is known for its zero-carbon emissions, high energy density of 142.35 kJ/kg, and wide applicability [3,4]. Electrochemical water splitting is the pivotal technology for green hydrogen production. It involves two key half-reactions: the hydrogen evolution reaction (HER) at the cathode and the oxygen evolution reaction (OER) at the anode [5,6]. However, its overall efficiency is significantly hampered by the sluggish OER. As a complex four-electron transfer process, the OER entails the formation of a thermodynamically stable O=O bond, a key step that requires overcoming a high energy barrier. This results in reaction kinetics that are typically 2–3 orders of magnitude slower than those of the HER [7,8]. Noble metal-based catalysts, such as RuO_2_ and IrO_2_, show exceptional OER catalytic activity. However, these materials tend to dissolve in the electrolyte under high potential. Moreover, their scarcity significantly hinders their large-scale implementation [9,10,11]. In contrast, transition metals (e.g., Fe-, Co-, and Ni-based compounds), with low cost and appropriate intrinsic activity, have become a research focus for OER catalysts, holding great promise to replace noble metal-based systems [12]. Among them, iron-based transition metals and their compounds exhibit excellent electrocatalytic water oxidation activity and stability in alkaline media, positioning them as promising substitute candidates for Ir- and Ru-based catalysts [13]. For instance, Liu et al. reported S-doped NiFe_2_O_4_ grown on iron foam (IF), which achieved an industrial-level current density of 500 mA cm^−2^ at a low overpotential of only 0.31 V [14]. Fan et al. reported that FeOOH-NiBDC on nickel foam (NF) reduced the overpotential to 0.32 V via finely tuning the dynamic interfacial ion balance [15]. Recently, the discovery of two-dimensional (2D) van der Waals (vdW) ferromagnetic materials (e.g., Fe_3_GeTe_2_, and Fe_3_GaTe_2_) has opened up new avenues for high-performance electrocatalysts [16,17]. These materials integrate the inherent advantages of 2D catalysts, such as an ultra-high surface area, abundant active sites, and suppressed charge recombination; their unique large spin moment characteristics can significantly promote the adsorption and activation of intermediates. Additionally, their intrinsic metallic properties endow them with fast carrier transport rates, a feature that is crucial for electrocatalysts [18].

Deiserath et al. first synthesized and characterized Fe_3_GeTe_2_, confirming its high magnetic anisotropy and distinct metallic properties [19]. Wang and Zhang’s teams later successfully prepared monolayer/few-layer Fe_3_GeTe_2_ nanosheets via mechanical exfoliation [20,21]. These ultra-thin samples exhibit robust out-of-plane ferromagnetic ordering and excellent metallic conductivity. Zhao et al. theoretically predicted that Fe_3_GeTe_2_ could serve as an excellent electrocatalyst, reducing the OER overpotential from 0.85 V to 0.3 V via surface hydroxyl self-reduction [22]. In practical applications, defects, particularly vacancies, are unavoidable. Surface vacancies in 2D materials can significantly enhance electrocatalytic activity by modulating the electronic structure (e.g., d-band center shift, charge redistribution) and coordination environment. For example, Zhang et al. found that oxygen-vacancy-rich CuMnO_2_ nanosheets promoted the CO_2_ reduction to ethylene [23]. Shao et al. showed that Mo vacancies enhanced HER performance in MoS_2_ [24]. Ma et al. introduced Te vacancies on the Fe_3_GeTe_2_ surface, exposing more basal plane active sites and improving nitrogen reduction activity [25].

This study employs first-principles density functional theory (DFT) calculations to investigate the effect of surface Te vacancies on the OER performance of the Fe_3_GeTe_2_ (001) basal plane. By introducing vacancies at different positions and concentrations, we systematically analyze the OER’s catalytic activity. Our results show that an appropriate Te vacancy concentration and configuration optimally enhances the OER activity of Fe_3_GeTe_2_ (001), reducing the overpotential to 0.34 V. This improvement arises from charge rearrangement around active sites and the enhanced adsorption of oxygen-containing intermediates, which lower the energy barrier of the OER’s rate-determining step. Our work provides theoretical insights into defect engineering in modulating the catalytic performance of 2D ferromagnetic materials and offers new perspectives for designing highly efficient and stable OER electrocatalysts.

## 2. Computational Model and Methods

**Figure 1:**
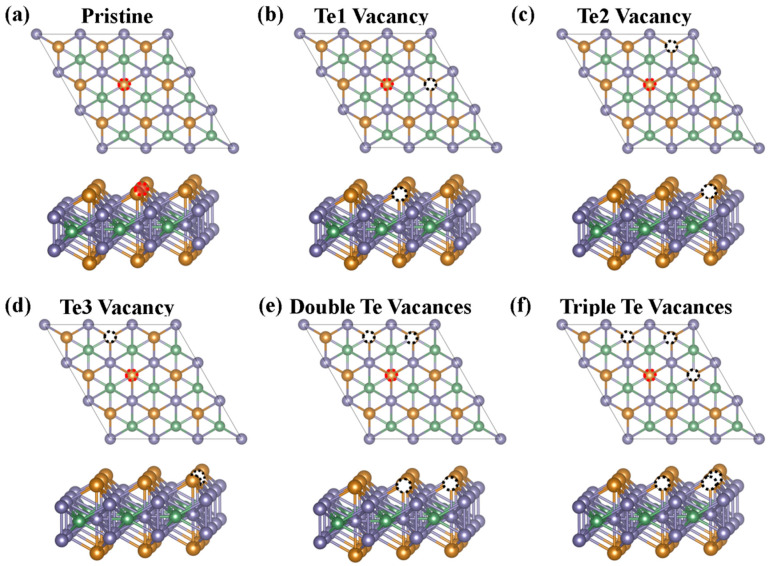
Top and side views of (**a**) the pristine Fe_3_GeTe_2_ (001) surface, the Fe_3_GeTe_2_ (001) defective surfaces with one vacancy (**b**) Te1, (**c**) Te2, (**d**) Te3, with a vacancy concentration of 5.56%, (**e**) double Te vacancies, with a vacancy concentration of 11.1%, and (**f**) triple Te vacancies, with a vacancy concentration of 16.7%, where Fe, Ge, and Te atoms are represented by blue, green, and orange spheres, respectively. The active sites and Te vacancies are indicated by red and white dashed circles, respectively.

All calculations were performed within the framework of density functional theory (DFT) using the VASP software. Different Te vacancy concentrations of 5.56%, 11.1%, and 16.7%, and different Te vacancy positions are considered in the Fe_3_GeTe_2_ (001) surface, as shown in Figure 1a. The (001) basal plane was derived by exfoliating the bulk Fe_3_GeTe_2_. We introduced the single- (Figure 1b–d), double- (Figure 1e), and triple (Figure 1f)-Te vacancy defects by removing surface-layer Te atoms from the surface layer, and added a 15 Å vacuum layer to eliminate periodic interactions. These surface Te vacancies can be experimentally realized through solid-state reactions, high-temperature annealing, or chemical reduction methods [26,27,28]. A 3 × 3 supercell was used for modeling, atomic positions were fully relaxed until the energy change between steps was <10^−5^ eV, and the force on atoms was <0.01 eV/Å. The plane-wave basis set cutoff energy was set to 400 eV, and the electron–ion interaction was described using the projector augmented wave (PAW) method. The exchange correlation effects were treated using local density approximation (LDA) [29]. For geometry optimization and electronic structure calculations, the Monkhorst–Pack k-point grids were set to 5 × 5 × 1 and 11 × 11 × 1, respectively. In our previous work, we had thoroughly verified the calculation method and finally confirmed that the LDA method was the most reliable, where the LDA-calculated structural parameters (*a* = *b* = 3.89 Å, *c* = 15.87 Å, and *m*_Fe_ = 1.33 *μ*_B_) agree well with the experimental values (*a* = *b* = 3.991 Å, *c* = 16.336 Å, and *m*_Fe_ = 1.2 *μ*_B_).

## 3. Results

### 3.1. Stability and Electronic Structure of Pristine and Te-Vacancy-Modified Fe_3_GeTe_2_ (001) Surfaces

As shown in Figure 1a, Fe_3_GeTe_2_ exhibits a typical hexagonal crystal structure with space group *P*6_3_/mmc. The structural features of Fe_3_Ge sublayers are sandwiched between two Te layers, with adjacent Te layers coupled by weak vdW forces. Notably, the crystal contains two distinct Fe sites: FeI atoms occupy the 4e Wyckoff sites, forming coordination bonds with surface Te atoms, while FeII atoms reside at 2c Wyckoff sites, and are coplanar with Ge atoms [30].

To further explore the effect of Te vacancies on the OER performance of the Fe_3_GeTe_2_ (001) basal plane, we constructed a series of defective surfaces with different Te vacancy concentrations, including single-Te-vacancy (Te1, Te2, and Te3), where the vacancy concentration is 5.56%; double-Te-vacancy, with a vacancy concentration of 11.1%; and triple-Te-vacancy configurations, where the vacancy concentration is 16.7%. The stability and feasibility of these defective surfaces were evaluated via defect formation energy *E_f_* calculations. The defect formation energy can be defined as follows:

(1) $$E_{f}=E_{\mathrm{defect}}-E_{\mathrm{perfect}}+\sum_{i}n_{i}u_{i}$$

**Figure 2:**
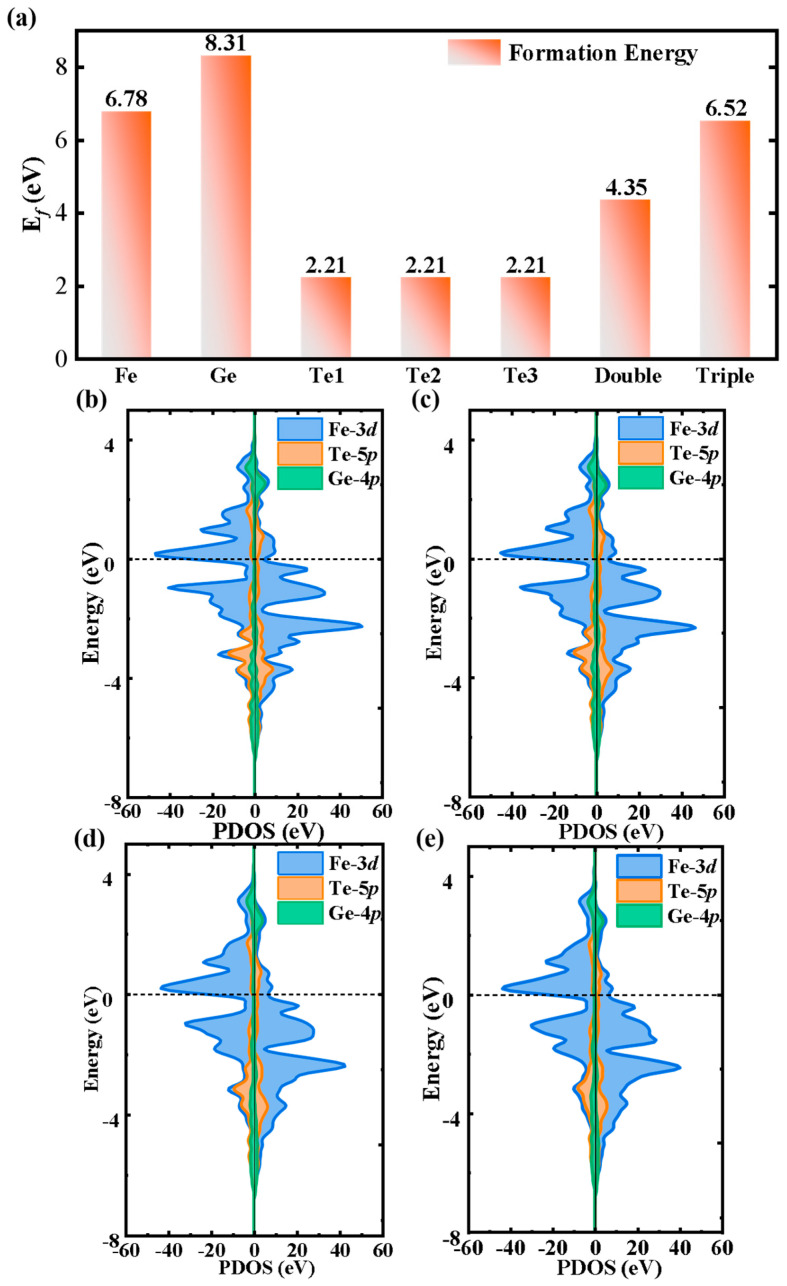
(**a**) The formation energies of different vacancy-defected Fe_3_GeTe_2_ (001) surfaces and spin-polarized projected density of states (PDOS) of (**b**) the pristine, (**c**) single-Te-vacancy-defected (Te1, Te2, and Te3), (**d**) double-Te-vacancy-defected, and (**e**) triple-Te-vacancy-defected Fe_3_GeTe_2_ (001) surface.

Here, *E*_defec_ represents the total energy of the Fe_3_GeTe_2_ (001) surface with Te vacancies, *E*_perfect_ denotes the total energy of the pristine (001) surface, *n_i_* is the number of removed Te atoms, and *u_i_* represents the chemical potential of Te atoms [31]. As shown in Figure 2a, the defect formation energies for single Fe and Ge vacancies on the (001) surface are 6.78 eV and 8.31 eV, respectively. By contrast, single-Te-vacancy configurations (Te1, Te2, and Te3) exhibit the lowest formation energy of 2.21 eV, indicating that Te vacancies are the most energetically favorable and structurally stable. While the defect formation energies of double-Te-vacancy and triple-Te-vacancy (001) surfaces are higher than that of the single-Te-vacancy configurations, they remain lower than those of the single-Fe or -Ge vacancies. Therefore, we selected these three defect configurations (varying Te vacancy positions and concentrations) for further investigation.

Figure 2b–e present the partial density of states (PDOS) for the pristine and Te-vacancy-defected Fe_3_GeTe_2_ (001) surface. The electronic structure of defected Fe_3_GeTe_2_ structures is similar to that of the pristine (001) surface, with asymmetric spin-up and spin-down channels, indicating that Te vacancies do not substantially alter the magnetic properties of the Fe_3_GeTe_2_. The energy bands are predominantly composed of Fe-3d orbitals near the Fermi level and the bands across the Fermi level, confirming that Te vacancies preserve the metallic nature of Fe_3_GeTe_2_. This metallic behavior enables high-speed electron transport, which is essential for high-performance electrocatalysts. Notably, since the Te atomic states are primarily distributed within the −4.5 to −2.5 eV energy range (well below the Fermi level), introducing Te vacancies negligibly modifies the material’s electronic transport properties.

### 3.2. Effect of Te Vacancy Sites on OER Performance of Fe_3_GeTe_2_ (001) Surface

In experimental studies, 1 M KOH of alkaline as the electrolyte is typically used for the OER process on the (001) surface. The material displays excellent stability at an applied potential of 2.5 V and outstanding OER activity in alkaline media [32]. Based on these findings, the electrochemical mechanism of the OER in alkaline media involves four electron-transfer steps:

(2) $$*{\;+\;\mathrm{OH}}^{-}\to {\mathrm{HO}}^{*}{+\;e}^{-}$$

(3) $${\mathrm{HO}}^{*}{+\mathrm{OH}}^{-}\to O^{*}{+H}_{2}{O+e}^{-}$$

(4) $$O^{*}{+\mathrm{OH}}^{-}\to {\mathrm{HOO}}^{*}{+e}^{-}$$

(5) $${\mathrm{HOO}}^{*}{+\mathrm{OH}}^{-}\to H_{2}{O+O}_{2}{+e}^{-}$$

Here, the asterisk denotes the active adsorption sites on the surface, while X^*^ represents oxygen-containing intermediates adsorbed at these active sites [33,34]. The Gibbs free energy change (ΔG) of the reaction can be defined as follows: ΔG = ΔE + ΔZPE − TΔS, where ΔE represents the total energy difference in the system before and after the reaction, ΔZPE denotes the zero-point energy correction term, and TΔS is the product of temperature and entropy change. In free energy calculations, an external bias U can be applied to each electron transfer step. The overall reaction occurs at the standard reduction potential of hydroxyl ions (U_0_ = 0.4 V), with the minimum free energy required to decompose four OH^−^ ions being 1.60 V [33]. Notably, the reaction kinetics are dictated by the step with the maximum free energy change (ΔG_max_). The overpotential (η) of the rate-determining step can be calculated using the formula $\eta \;=\;\frac{1}{e}∆G_{max}\;-\;U_{0}$ [34]. For the Fe_3_GeTe_2_ (001) basal plane, the surface Te atoms serve as active sites, and the calculated overpotential is η = 0.85 V, consistent with previous theoretical results [22].

**Figure 3:**
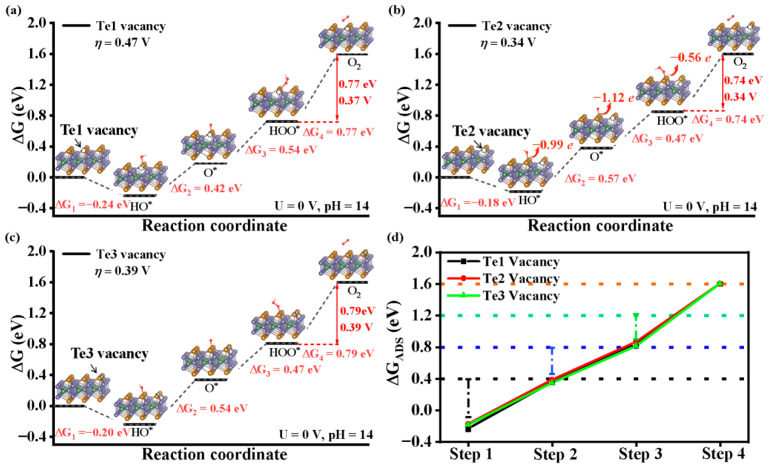
The Gibbs free energy of the OER process on the Fe_3_GeTe_2_ (001) surface with (**a**) Te1 vacancy, (**b**) Te2 vacancy, and (**c**) Te3 vacancy under the conditions of U = 0 V and pH = 14. (**d**) Comparison of the Gibbs free energies of oxygen-containing intermediates in each step with the ideal values.

Figure 3a–c present the Gibbs free energy diagrams for the OER process on Fe_3_GeTe_2_ (001) surfaces with single-Te vacancies (Te1, Te2, and Te3, respectively). The Gibbs free energy values for each reaction step are as follows: Te1 vacancy surface: −0.24, 0.42, 0.54, and 0.77 eV; Te2 vacancy surface: −0.18, 0.57, 0.47, and 0.74 eV; and Te3 vacancy surface: −0.20, 0.54, 0.47, and 0.79 eV. The rate-determining step consistently occurs at the fourth step (HOO* → O_2_), with corresponding overpotentials η of 0.37 V, 0.34 V, and 0.39 V for Te1, Te2, and Te3 vacancy surfaces, respectively. Although the vacancy concentrations and formation energies of Te1, Te2, and Te3 in Fe_3_GeTe_2_ are identical at 2.21 eV, differences in their crystal structure persist. There are slight variations in bond lengths and bond angles. It is these structural disparities that result in differences in the binding strength between active sites and oxygen-containing intermediates. Consequently, the Gibbs free energy at each electronic step of the oxygen evolution has variations in the OER overpotential. Compared to the pristine Fe_3_GeTe_2_ (001) surface (η = 0.85 V), Te vacancies significantly enhance the OER catalytic activity and substantially reduce the overpotential. Moreover, this lower potential can rival the ideal OER performance of IrO_2_ (0.56 V) [35].

We further calculate the free energies of the oxygen-containing intermediates and compare them with the ideal values, which are defined as follows:

(6) $$\Delta G_{\mathrm{HO}*}=\Delta G_{1}$$

(7) $$\Delta G_{O*}=\Delta G_{1}+\Delta G_{2}$$

(8) $$\Delta G_{\mathrm{HOO}*}=\Delta G_{1}+\Delta G_{2}+\Delta G_{3}$$

(9) $$\Delta G_{O_{2}}=\Delta G_{1}+\Delta G_{2}+\Delta G_{3}+\Delta G_{4}$$

Here, ΔG_1_, ΔG_2_, ΔG_3_, and ΔG_4_ represent the Gibbs free energies for the first to fourth reaction steps, respectively [36,37]. As shown in Figure 3d, the dashed lines indicate the ideal values for Δ*G*_HO*_, Δ*G*_O*,_ Δ*G*_HOO*_, and Δ*G*_O2_, which are 0.4, 0.8, 1.2, and 1.6 eV, respectively [27]. The closer the free energy values are to these ideal benchmarks, the better the binding between the active site and the oxygen-containing intermediates. If the free energy is higher than the ideal value, it indicates stronger adsorption. Conversely, a lower free energy suggests stronger binding [38,39].

The introduction of Te vacancies significantly facilitates the adsorption of oxygen-containing intermediates (HO*, O*, and HOO*) on the Fe_3_GeTe_2_ surface, especially for HOO* intermediates, which poorly adsorb on the pristine surface. Te vacancies improve HOO* adsorption, reducing the adsorption free energy to approach the ideal value. This is verified via Bader charge analysis, which shows enhanced charge transfer from Te active sites to oxygen-containing intermediates (HO*, O*, and HOO*) due to Te vacancies. For instance, on the Te2-vacancy-defected (001) surface, the active Te site transfers 0.99, 1.12, and 0.56 e to the HO*, O*, and HOO* intermediates, respectively. In contrast, in the absence of Te vacancies, the active Te sites transfer 0.59, 0.66, and 0.2 e to HO*, O*, and HOO*, respectively. Therefore, the introduction of Te vacancies is beneficial for enhancing the OER activity of Fe_3_GeTe_2_, and different Te vacancy positions exhibit nearly identical effects on intermediate adsorption and OER performance improvement for the Fe_3_GeTe_2_ (001) surface.

### 3.3. Effect of Te Vacancy Concentration on OER Performance of Fe_3_GeTe_2_ (001) Surface

**Figure 4:**
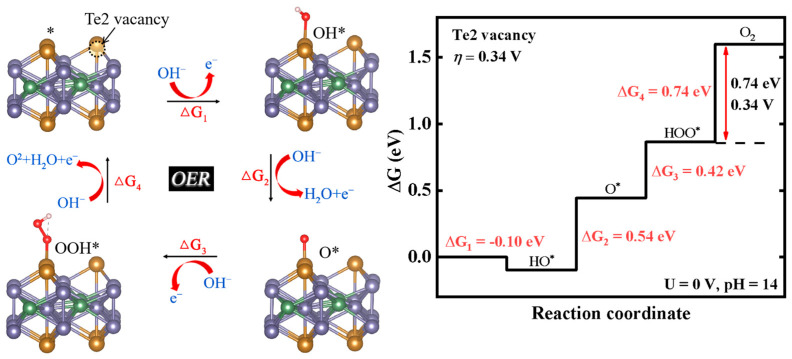
The OER pathway on the 2 × 2 Fe_3_GeTe_2_ surface with a Te vacancy on the surface (vacancy concentration: 12.5%), and free energy diagram of the OER process at U = 0 V and pH = 14.

Furthermore, we reconstructed a 2 × 2 Fe_3_GeTe_2_ supercell containing a single Te vacancy (vacancy concentration: 12.5%), as shown in Figure 4. Surprisingly, there is a low overpotential of 0.34 V during the OER process. It is worth noting that despite the difference in concentrations, both systems involve only one Te vacancy, and their OER processes are similar, with identical overpotentials of 0.34 V. By contrast, at the same Te vacancy concentration (e.g., 5.56% as shown in Figure 3), the overpotentials are different.

**Figure 5:**
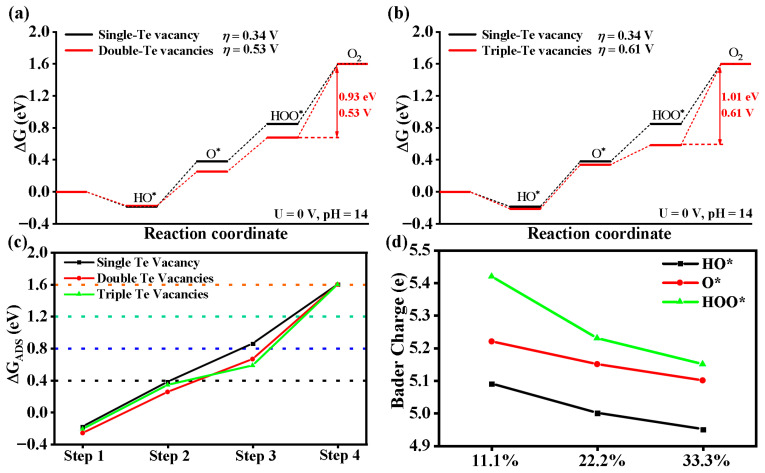
The comparison of Gibbs free energies for the OER process on Fe_3_GeTe_2_ (001) surfaces with single-Te (**a**) double-Te vacancies and (**b**) triple-Te vacancies under the conditions of U = 0 V and pH = 14. (**c**) Comparison of the Gibbs free energies of oxygen-containing intermediates at each step of the Te-vacancy-defected surface at different concentrations with the ideal values. (**d**) Bader charges on the active-site Te atoms on different Te-vacancy-defected surfaces.

Is the OER process related to the concentration? We systematically investigate the effect of vacancy concentration on the Fe_3_GeTe_2_ (001) surface by constructing defective systems with single-, double-, and triple-Te vacancies, corresponding to vacancy concentrations of 5.56%, 11.1%, and 16.7%, respectively, in Figure 5. The Gibbs free energy of each oxygen-containing intermediate decreases as the vacancy concentration increases, with the adsorption of HOO* particularly enhanced. The rate-determining step remains the fourth step (HOO* → O_2_), with overpotentials *η* of 0.53 V and 0.61 V for double- and triple-Te-vacancy systems, respectively, both significantly higher than the single-vacancy system’s 0.34 V, but lower than that of the pristine Fe_3_GeTe_2_. For theses double- and triple-vacancy models, Te vacancies are located at adjacent sites, and the OER process is affected not only by the increased vacancy concentration, but also by Te vacancies clustering, that is, Te vacancies’ configuration also influences the OER process.

Figure 5c compares the free energies with the ideal values, and it is found that in double- and triple-Te vacancy systems, the HOO* adsorption free energy deviates more from the ideal value. This excessive enhancement of HOO* adsorption impedes the O_2_ desorption, increasing the reaction energy barrier. Bader charge analysis (Figure 5d) reveals a negative correlation between the Te vacancy concentration and charge density at active Te sites: higher vacancy concentrations lead to a gradual decrease in electron density at catalytic centers, directly correlating with reduced OER performance. For example, when the vacancy concentration is 5.56%, the charge quantities of oxygen-containing intermediates (HO*, O*, and HOO^*^) are 5.09, 5.22, and 5.42 e, respectively, with an overpotential (η) of 0.34 V. At concentrations of 11.1% and 16.7%, the charge quantities of the oxygen-containing intermediates (HO*, O*, and HOO*) decrease to 5.0, 5.15, and 5.23 e and 4.95, 5.1, and 5.15 e, respectively, with the overpotentials (η) of 0.53 V and 0.61 V. Therefore, changing the Te vacancy concentration and configuration has a great influence on the OER activity on the Fe_3_GeTe_2_ (001) surface. An optimal Te vacancy concentration and configuration can effectively regulate the charge distribution at active sites, reduce the OER overpotential of the rate-determining step, and enhance the OER catalytic activity of Fe_3_GeTe_2_.

## 4. Conclusions

This work systematically investigates the effect of Te vacancy defects on the oxygen evolution reaction (OER)’s catalytic performance of the Fe_3_GeTe_2_ (001) basal plane using first-principles density functional theory (DFT) calculations. Defect formation energy calculations show that Te-vacancy-defected surfaces have good stability. The introduction of Te vacancies significantly enhances the OER activity of Fe_3_GeTe_2_, reducing the overpotential from 0.85 V (pristine state) to 0.34 V, which is comparable to IrO_2_ (0.56 V). Notably, Te vacancies at different positions (Te1, Te2, and Te3) exhibit highly consistent OER kinetics and intermediate adsorption, indicating that the Te vacancy position has negligible effects on the OER performance of the Fe_3_GeTe_2_ (001) surface. As the vacancy concentration increases from 5.56% (single vacancy) to 16.7% (triple vacancies), the Bader charge analysis reveals a decreasing trend in charge density at active-site Te atoms, leading to an excessive adsorption of the HOO^*^ intermediate. This hinders O_2_ desorption, increasing the OER overpotential from 0.34 V to 0.61 V and degrading catalytic activity. Therefore, an appropriate Te vacancy concentration and configuration optimize charge distribution at active sites, reducing the energy barrier of the rate-determining step and enhancing catalytic efficiency. This study elucidates the microscopic mechanism of defect engineering in regulating the catalytic performance of 2D ferromagnetic materials and provides theoretical insights for the development of highly active and stable OER electrocatalysts.

## Data Availability

The data that support the findings of this study are available within the article.
